# miR-34a: a new player in the regulation of T cell function by modulation of NF-κB signaling

**DOI:** 10.1038/s41419-018-1295-1

**Published:** 2019-01-18

**Authors:** Martin Hart, Barbara Walch-Rückheim, Kim S. Friedmann, Stefanie Rheinheimer, Tanja Tänzer, Birgit Glombitza, Martina Sester, Hans-Peter Lenhof, Markus Hoth, Eva C. Schwarz, Andreas Keller, Eckart Meese

**Affiliations:** 1 Institute of Human Genetics, Saarland University, 66421 Homburg, Germany; 2 Institute of Virology and Center of Human and Molecular Biology, Saarland University Medical School, 66421 Homburg, Germany; 3 Biophysics, Center for Integrative Physiology and Molecular Medicine, School of Medicine, Saarland University, 66421 Homburg, Germany; 4 Department of Transplant and Infection Immunology, Saarland University, 66421 Homburg, Germany; 5 Center for Bioinformatics, Saarland University, 66123 Saarbrücken, Germany; 6 Saarland University, 66123 Saarbrücken, Germany

## Abstract

NF-κB functions as modulator of T cell receptor-mediated signaling and transcriptional regulator of miR-34a. Our in silico analysis revealed that miR-34a impacts the NF-κB signalosome with miR-34a binding sites in 14 key members of the NF-κB signaling pathway. Functional analysis identified five target genes of miR-34a including *PLCG1*, *CD3E*, *PIK3CB*, *TAB2*, and *NFΚBIA*. Overexpression of miR-34a in CD4^+^ and CD8^+^ T cells led to a significant decrease of NFΚBIA as the most downstream cytoplasmic NF-κB member, a reduced cell surface abundance of TCRA and CD3E, and to a reduction of T cell killing capacity. Inhibition of miR-34a caused an increase of NFΚBIA, TCRA, and CD3E. Notably, activation of CD4^+^ and CD8^+^ T cells entrails a gradual increase of miR-34a. Our results lend further support to a model with miR-34a as a central NF-κB regulator in T cells.

## Introduction

Toward a deeper understanding of the immune response, it is crucial to dissect the molecular mechanisms regulating the activity of immune cells. MicroRNAs (miRNAs, miRs) play a central role in the regulation of development and homeostasis, particularly in T cell differentiation^1^. MiRNAs are small non coding RNAs of—21–24 nucleotides in length that regulate gene expression post-transcriptionally^2^. On the cellular level, miRNAs control various processes including differentiation, signal transduction, and apoptosis^3–5^. We previously reported aberrantly expressed miRNAs in whole blood from patients with different tumor identities^6–8^. Analysis of the miRNA expression in different blood cell subpopulations showed significant overexpression of miR-34a in CD3^+^ T cells of lung cancer patients^9^. MiRNA-34a directly targets five PKC-isozymes^10^ including PRKCQ (protein kinase C theta) which controls T cell functions by regulating signaling pathways leading to activation of nuclear factor kB (NF-κB)^11^. NF-κB is a major modulator of T cell receptor-mediated signaling controlling adaptive immune responses by modulating T cell fate^12,13^. Previously we identified *TCRA* (T-cell receptor alpha locus) as direct target gene of miR-34a. Notably two non-canonical 5-mer sites of miR-34a in the 3′ UTR of *TCRA* had a significantly stronger impact on its posttranscriptional regulation than the canonical binding sites^14^. To further investigate the impact of miRNA-34a on the NF-κB signalosome, we analyzed key players in NF-κB signaling for posttranscriptional regulation by this miRNA. Within the NF-κB signaling cascade we identified miR-34a binding sites in the 3′UTRs of 14 key modulators including, *PLCG1* (phospholipase C gamma 1), *CD3E* (CD3e molecule), *PIK3CB* (phosphatidylinositol-4,5-bisphosphate 3-kinase catalytic subunit beta), *TAB2* (TGF-beta activated kinase 1/MAP3K7 binding protein 2), and *NFΚBIA* (NFKB inhibitor alpha), with the latter also showing a significantly reduced luciferase activity upon co-transfection with a 3′ UTR reporter vector and a miR-34a expression plasmid. While overexpression of miR-34a led to a decrease of endogenous NFΚBIA as the most downstream cytoplasmic NF-κB pathway member, transfection of anti-miR-34a caused a significant increase of the NFΚBIA protein level in primary CD4^+^ and CD8^+^ T cells. As for the upstream effect, ectopic expression of miR-34a significantly decreased cell surface expression of TCRA and CD3E in CD4^+^ and CD8^+^ T cells. Inhibition of miR-34a resulted in increased cell surface levels of CD3E and TCRA in CD4^+^ T cells and of TCRA in CD8^+^ T cells. CD8^+^ T cells overexpressing miR-34a displayed a reduced target cell killing 30 and 50 h after transfection. We propose a model on how miR-34 likely acts on the NF-κB pathway in T cells.

## Methods and materials

### Cell lines, tissue culture

The human HEK 293T and Jurkat cells were purchased from the German collection of microorganisms and cell cultures (DSMZ) and authenticated using STR DNA typing. HEK 293T cells were cultured in DMEM (Life Technologies GmbH, Darmstadt, Germany) supplemented with 10% fetal bovine serum (Biochrom GmbH, Berlin, Germany), Penicillin (100 U/mL), Streptomycin (100 μg/mL). Cells were passaged for less than 6 months after receipt. Jurkat, T2, and lymphoblastoid cells were cultured in RPMI1640 (Life Technologies GmbH, Darmstadt, Germany) supplemented with 10% fetal bovine serum (Biochrom GmbH, Berlin, Germany), Penicillin (100 U/mL), Streptomycin (100 μg/mL). Cells were passaged for less than 6 months after receipt.

### CD4^+^ and CD8^+^ T cells from healthy donors

CD4^+^ T cells were isolated by negative selection from freshly obtained PBMC using human CD4^+^ T cell isolation kit (Miltenyi Biotech, Bergisch Gladbach, Germany). Purity was confirmed with CD4-FITC (Cat# 555346, BD Bioscience) and analyzed by flow cytometry. CD8^+^ T cells were isolated by negative selection from freshly obtained PBMC using human CD8^+^ T cell isolation kit (Miltenyi Biotech, Bergisch Gladbach, Germany). Purity was confirmed with CD8-FITC (Cat# 555366, BD Bioscience) and analyzed by flow cytometry. Cells were cultured in RPMI 1640 medium (Sigma) supplemented with 10% heat-inactivated endotoxin-tested FCS (Biochrom GmbH, Berlin, Germany).

### Generation and expansion of MART1-specific CD8^+^ T cell clones

MART1 (melanoma antigen recognized by T cells 1)-specific CD8^+^ T cell clones were generated as described before^15^. In brief, monocytes were isolated from PBMC and stimulated with IL-4 and GM-CSF for 72 h in Cellgro DC medium (CellGenix) supplemented with 1% human serum (Sigma Aldrich) to generate immature DC (dendritic cells). Maturation of DC was induced by GM-CSF, IL-4, LPS, IFNγ and MART1 peptide for 16 h at 37 °C. Autologous naïve CD8^+^ T cells were isolated from frozen PBMC. Mature DC (irradiated at 30 Gy) and naïve CD8^+^ T cells were cocultured for 10 days in Cellgro DC medium supplemented with 5% human serum. IL-21 was added at day 1, IL-7 and IL-15 at days 3, 5, and 7. After 10 days MART1-loaded, autologous PBMC (irradiated at 30 Gy) were cocultured with CD8^+^ T cells for 6 h. Antigen-specific CD8^+^ T cells were isolated using IFN-γ Secretion Assay. Cells were seeded with 1 cell/well (200 µL/well) in RPMI1640 supplemented with 10% human serum, Penicillin-Streptomycin (100U/mL–100µg/mL, Sigma Aldrich), 30 ng/mL anti-CD3 antibody (clone:OKT3), 50U/mL IL-2, 5 × 10^4^ allogenous PBMC/well (irradiated at 30 Gy) and 5 × 10^4^/well of a lymphoblastoid cell line (irradiated at 120 Gy) in 96-well U-bottom plates. After 7 days, 50 µL of RPMI1640 supplemented with 10% human serum, Penicillin–Streptomycin and 250 U/mL IL-2 were added to each well and incubated for another week. Proliferating CD8^+^ T cells clones were transferred in a 25 cm^2^ cell culture flask containing 25 × 10^6^ PBMC (irradiated at 30 Gy) and 5 × 10^6^ cells of a lymphoblastoid cell line (irradiated at 120 Gy) in 20 ml RPMI1640 supplemented with 10% fetal bovine serum, Penicillin-Streptomycin for expansion. At days 1, 3, 5, 8, and 11 1200 U IL-2 and 40 ng IL-15 were added. Antigen specificity was assessed using MART1-specific dextramers in flow cytometry. Antigen-specific clones were frozen in aliquots and further experiments were performed at days 11–14 of expansion.

### Cloning of reporter constructs

The 3′UTRs of NFΚBIA, RELA, cREL, IKBKB, IKBKG, TAB1, TAB2, TAK1, TRAF2, BCL10, PIK3CB, MALT1, PLCG1, and CD3E were cloned into the pMIR-RNL-TK vector that was described in Beitzinger et al. using the SpeI, SacI, or NaeI restriction sites^16^. The nucleotides 743–1228 and 3578–4120 of the cREL 3′UTR (NM_002908.3), nucleotides 46–744 of the RELA 3′UTR (NM_021975.3), nucleotides 1–433 of the NFΚBIA 3ʹUTR (NM_020529.2), nucleotides 1–520 of the IKBKG 3ʹUTR (NM_001099857.2), nucleotides 189–900 of the IKBKB 3ʹUTR (NM_001556.2), nucleotides 829–982 of the TAK1 3ʹUTR (NM_003298.4), nucleotides 144–900 of the TAB1 3ʹUTR (NM_006116.2), nucleotides 102–1009 of the TAB2 3ʹUTR (NM_015093.5), nucleotides 1–514 of the TRAF2 3ʹUTR (NM_021138.3), nucleotides 308–1401 of the BCL10 3ʹUTR (NM_003921.4), nucleotides 1062–2243 of the MALT1 3ʹUTR (NM_006785.3), nucleotides 136–1200 of the PLCG1 3ʹUTR (NM_002660.2), nucleotides 1436–2137 of the PIK3CB 3ʹUTR (NM_006219.2) and nucleotides 1–573 of the CD3E 3ʹUTR (NM_000733.3) were amplified by PCR using specific primers (Supplementary Table S1) from Jurkat cDNA. All hsa-miR-34a-5p target sites were mutated by site-directed mutagenesis using the QuickChange II Site-Directed Mutagenesis Kit (Agilent Technologies, Santa Clara, United States) with specific primers (Supplementary Table S1).

### Dual luciferase reporter assays

6.5 × 10^4^ HEK 293T cells were seeded out per well of a 24-well plate. The next day the cells were transfected with 0.8 μg miRNA expression plasmid or control plasmid and 0.2 μg reporter vector with 3’UTR or 0.2 μg empty control reporter vector in the appropriate combinations using PolyFect transfection reagent according to the manufacturer’s protocol (Qiagen, Hilden, Germany). Forty-eight hours after transfection the cells were lysed and measured corresponding to the Dual Luciferase® Reporter Assay System protocol (Promega, Mannheim, Germany). All luciferase reporter assays were carried out in duplicates and were repeated four times.

### Overexpression of miR-34a in Jurkat, CD4^+^ and CD8^+^ T cells and western blot

For western blot analysis, 1 × 10^6^ CD4^+^ or CD8^+^ T cells per well were seeded out in 12-well plates. 2.5 × 10^5^ Jurkat cells were seeded out per well of a 6-well plate. Subsequently they were transfected either with the allstars negative control (ANC) and with hsa-miR-34a-5p miScript miRNA Mimic (MIMAT0000255: 5′UGGCAGUGUCUUAGCUGGUUGU), respectively or with miScript Inhibitor Negative Control and Anti-hsa-miR-34a-5p miScript miRNA Inhibitor: (MIMAT0000255: 5′UGGCAGUGUCUUAGCUGGUUGU) according to the HiPerFect transfection reagent protocol (Qiagen, Hilden, Germany). Forty-eight hours after transfection, the cells were lysed with 2× lysis buffer (130 mM Tris/HCl, 6% SDS, 10% 3-Mercapto-1,2-propandiol, 10% glycerol) and sonicated 3 times for 2 s. Fifteen micrograms of the whole protein extracts were separated using a Mini-Protean^®^ TGX Stain-Free^TM^ Precast Gel (Bio-Rad Laboratories Inc., Hercules, California, USA) and electroblotted on a nitrocellulose membrane (Whatman, GE Healthcare, Freiburg, Germany). The detection of NFΚBIA was carried out with a monoclonal antibody against NFΚBIA (Cat# 4814, Cell Signaling Technology, Danvers, United States). GAPDH served as loading control and was detected with a monoclonal antibody against GAPDH (Cat# 2118, Cell Signaling Technology, Danvers, United States). All secondary antibodies were purchased from Sigma Aldrich (Sigma Aldrich, Munich, Germany).

### Overexpression of miR-34a in MART1-specific CD8^+^ T cells

MART1-specific CD8^+^ T cells were transfected at day 11 of expansion. 6 × 10^6^ cells were transfected either with 8 μL 20 μM solution of allstars negative control (ANC) or hsa-miR-34a-5p miScript miRNA Mimic, respectively, using P3 Primary Cell 4D-Nucleofector X Kit (Lonza). Thirty hours after transfection cells were washed and resuspended in AIMV medium (Thermo Fisher Scientific) supplemented with 10% fetal bovine serum, 50 U/mL IL-2 and 5 ng/mL IL-15. 30 and 50 h after transfection cells were used to perform real-time killing assays.

### Antibodies and flow cytometry

The surface antigens CD4, CD8, CD3E, and TCR alpha were stained with the following fluorescent labeled antibodies: anti CD4-PE (Cat# 555347, BD Biscience), anti CD8-PE (Cat# 130-091-084, Miltenyi Biotech), anti-CD3E (BD Biosciences, Cat# 561806) and anti TCRA alpha/beta (Thermo Fisher Scientific, Cat# 17-9986-41) for 30 min at 4 °C. Cells were fixed in 1% paraformaldehyde and analyzed by flow cytometry (FACS Canto II, BD Biosciences).

### Real-time killing assay

Killing of T2 cells by MART1-specific CD8^+^ T cell clones was measured by a time-resolved, real-time killing assay over a time period of 4 h. The real-time killing assay was carried out as described before^17^. In brief, T2 cells were loaded with 2.5 µg MART1-specific peptide in 500 µL AIMV medium supplemented with 10% fetal bovine serum for 90 min at 37 °C and 5% CO_2_. 0.5 × 10^6^MART1-T2 cells were loaded with calcein-AM (500 nM) in AIMV medium supplemented with 10 mM HEPES (AIMV*) for 15 min at room temperature. Cells were centrifuged at 200×*g* for 5 min resuspended in 4 mL AIMV*. 200 µL per well (25 × 103 target cells) were plated in a black, clear-bottom, 96-well plate (353219, Corning). Cells were settled down for at least 15 min at room temperature. CD8^+^ T cell clones were added slowly at an effector to target ratio of 2:1. Measurement was started immediately in a GENios Pro plate reader (Tecan) at 37 °C every 10 min for 4 h using bottom reading mode. The fraction of killed cells is then calculated for each time point by the equation:

$${{\mathrm {target}}\,{\mathrm {lysis}} \left( \% \right) = \left( {{\mathrm {Fexp}}- {{\mathrm {Flive}}}^\ast I} \right)/\left( {\left( {{\mathrm {Flysed}} - {\mathrm {Flive}}} \right)^\ast I} \right)^\ast 100}$$

(Flive: Fluorescence of target cells only; Flysed: fluorescence of lysed target cells only; Fexp: Fluorescence of the experimental well; *I* (Index): Fexp(at timepoint 0)/Flive (at timepoint 0) All fluorescence values are subtracted by the corresponding medium controls)

### Activation of CD4^+^ and CD8^+^ cells and western blot

1 × 10^6^ freshly isolated CD4^+^ or CD8^+^ T cells per 12-well were stimulated with the T Cell Activation/Expansion Kit (bead-to-cell ratio 1:4, Cat# 130-091-441, Miltenyi Biotech) for 4 h.

Nuclear and cytoplasmic extracts were prepared according to ref. ^18^ with minor modifications^19^. Briefly, T cells were harvested by centrifugation and washed with cold phosphate-buffered saline (PBS). The pellet was resuspended in 150 mL of buffer A (10 mmol/L HEPES, pH 7.9, 10 mmol/L KCl, 0.1 mmol/L EDTA, pH 8.0, 0.1 mmol/L EGTA, 1 mmol/L dithiothreitol (DTT), 100 μg/mL phenylmethylsulfonyl fluoride (PMSF), 1 μg/mL aprotinin, 2 μg/mL leupeptin, 100 μg/mL Pefabloc, and 100 μg/mL chymostatin) by gentle pipetting and incubated on ice for 15 min. 10 μL of 10% Nonidet-P-40 solution (Sigma) was added and cells were vigorously mixed for 10 s before centrifugation. The supernatant containing the cytoplasmic proteins was transferred to another tube. Pelleted nuclei were resuspended in 50 μL of buffer C (25% glycerol, 20 mmol/L HEPES, pH 7.9, 0.4 mol/L NaCl, 1 mmol/L EDTA, pH 8.0, 1 mmol/L EGTA, 1 mmol/L DTT, 100 μg/mL PMSF, 1 μg/mL aprotinin, 2 μg/mL leupeptin, 100 μg/mL Pefabloc, and 100 μg/mL chymostatin) and mixed at 4 °C for 20 min. The nuclei were centrifuged for 10 min at 13,000 rpm and supernatants containing the nuclear proteins were stored at −80 °C.

15 µg of the cytoplasm or nucleus extracts were separated using a Mini-Protean^®^ TGX Stain-Free^TM^ Precast Gel (Bio-Rad Laboratories Inc., Hercules, California, USA) and electroblotted on a nitrocellulose membrane (Whatman, GE Healthcare, Freiburg, Germany). The detection of NFΚBIA was carried out with a monoclonal antibody against NFΚBIA (Cat# 4814, Cell Signaling Technology, Danvers, United States) or a monoclonal antibody against p65 (Cat# 8242, Cell Signaling Technology, Danvers, United States), respectively. GAPDH served as loading control and was detected with a monoclonal antibody against GAPDH (Cat# 2118, Cell Signaling Technology, Danvers, United States). All secondary antibodies were purchased from Sigma Aldrich (Sigma Aldrich, Munich, Germany).

### RNA isolation and quantitative real-time PCR (qRT-PCR)

Seven hours after activation of 1 × 10^6^ CD4^+^ or CD8^+^ T cells or 48 h after transfection of 1 × 10^6^ CD4^+^ or CD8^+^ T cells with the allstars negative control (ANC) and with hsa-miR-34a-5p miScript miRNA Mimic (MIMAT0000255: 5′UGGCAGUGUCUUAGCUGGUUGU), cells were lysed using Qiazol (Qiagen, Hilden, Germany) and the total RNA was isolated according to the protocol of the miRNAeasy Micro KIT (Qiagen, Hilden, Germany). The expression of hsa-miR-34a-5p was analyzed by qRT-PCR using the miScript PCR System (Qiagen, Hilden, Germany) and the StepOnePlus Real-Time PCR System (Applied Biosystems, Foster City, United States) corresponding to the manufacturer’s protocol. In brief 150 ng total RNA was reverse transcribed into cDNA using the miScript RT II Kit with the miScript HiSpec Buffer (Qiagen, Hilden, Germany). RNU48 or served as endogenous control.

### Statistical analysis and quantification

The statistical analysis of the dual luciferase assays, the western blots and the FACS experiments was conducted with SigmaPlot 10 (Systat, Chicago, USA) applying Student’s *t*-test. The densitometric analysis of western blots was carried out with Image Lab Software Version 5.2.1 (Bio-Rad Laboratories Inc., Hercules, California, USA). Data are statistically significant when *p* < 0.05 by Student’s *t* test. In figures, asterisks correspond to the statistical significance as calculated by Student’s *t*-test: * = 0.01 < *p* ≤ 0.05; ** = 0.001 < *p* ≤ 0.01; *** = *p* < 0.001.

## Results

### Target prediction and validation of *PLCG1*, *CD3E*, *PIK3CB*, *TAB2*, and *NFΚBIA* as direct target genes of miR-34a by dual luciferase assay

**Fig. 1:**
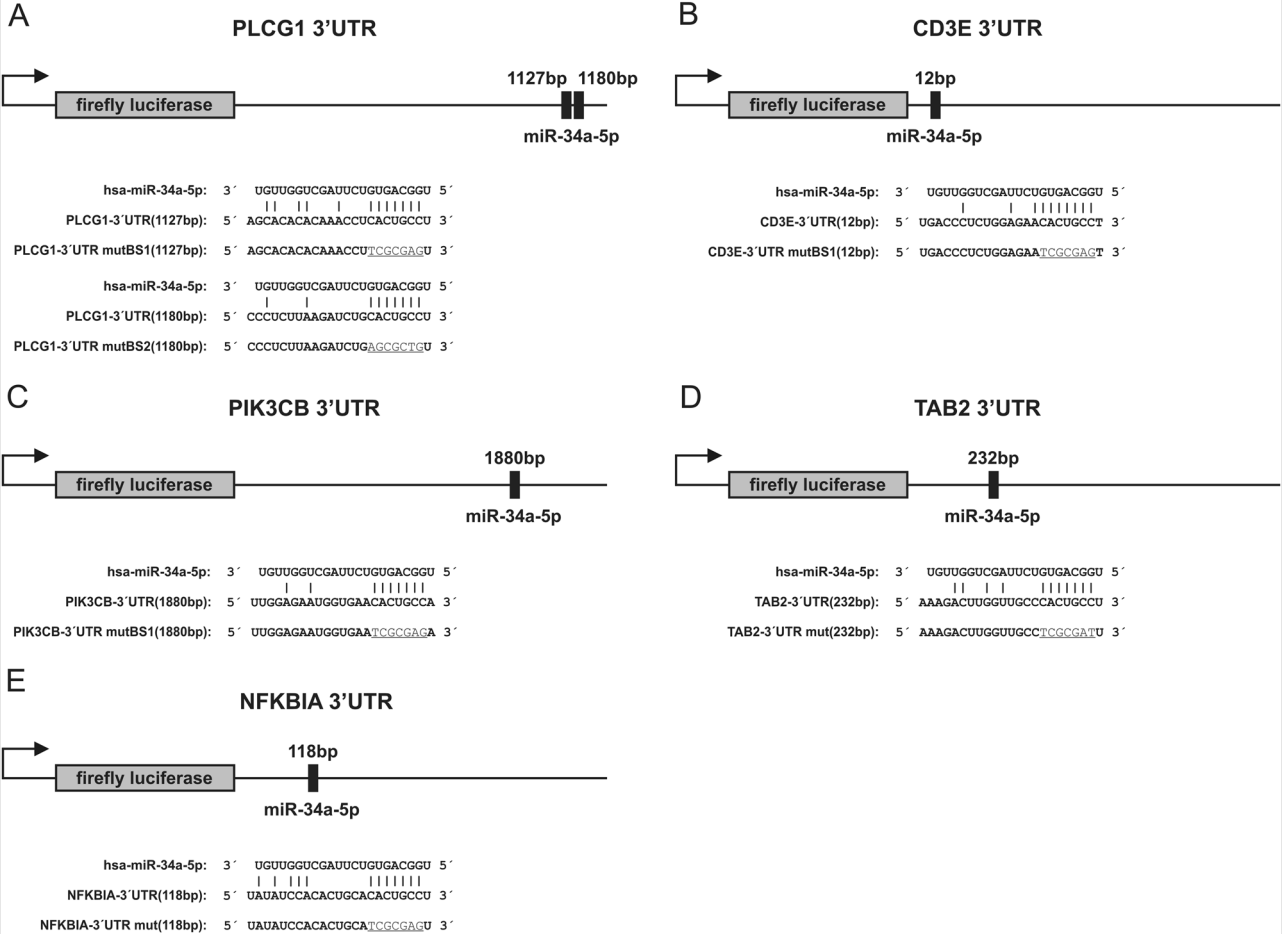
Schematic representation of reporter gene constructs. The position of the predicted miR-34a-5p binding sites in the respective 3′UTR reporter constructs and additionally the sequences of the binding sites of miR-34a-5p in the different 3′UTRs as well as the mutated binding sites (underlined) are shown. **a** *PLCG1*-3′UTR reporter vector, **b** *CD3E*-3’UTR reporter vector, **c** *PIK3CB*-3′UTR reporter vector, **d** *TAB2*-3′UTR reporter vector, **f** *NFΚBIA*-3′UTR reporter vector

**Fig. 2:**
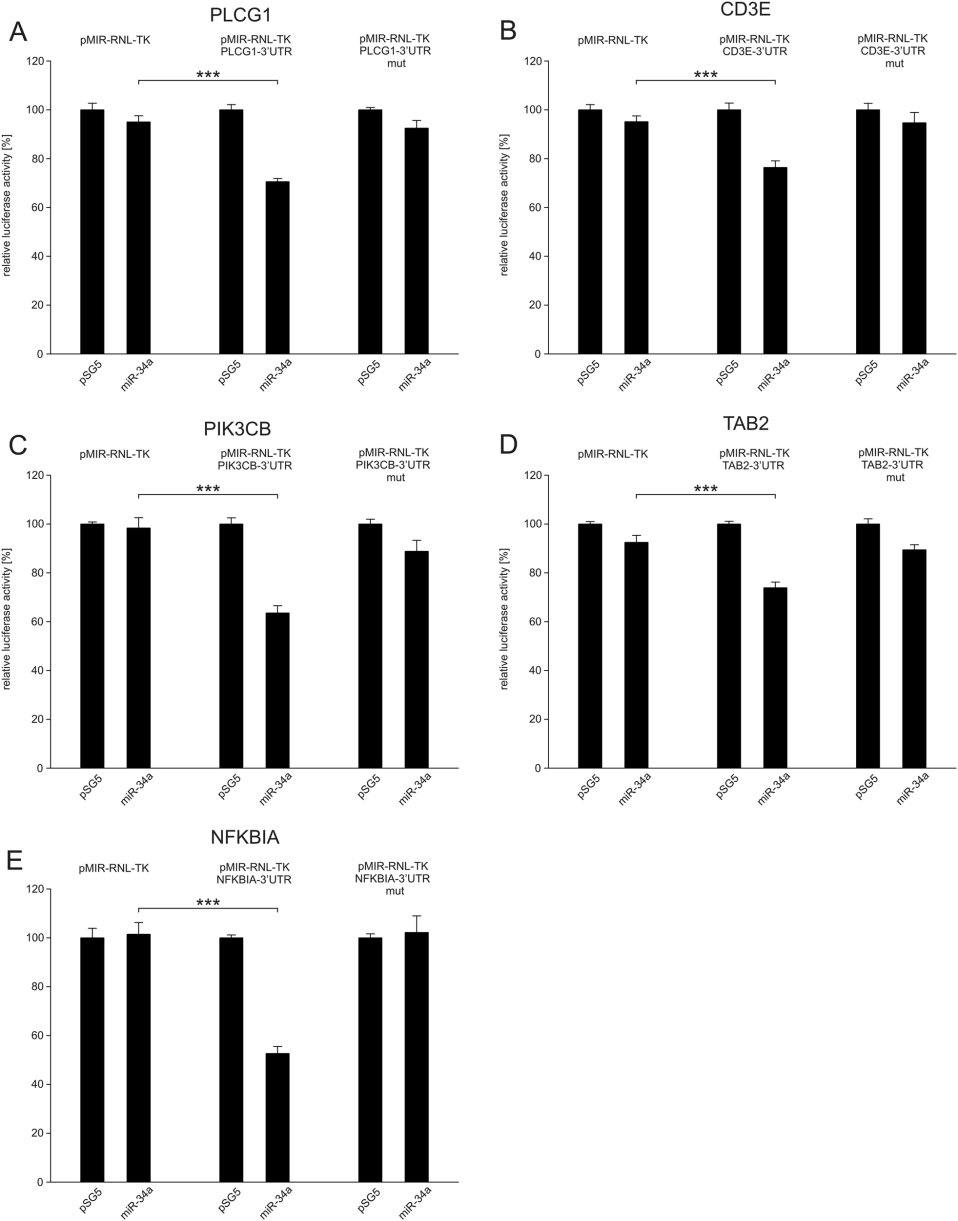
Dual luciferase reporter assays of *PLCG1*, *CD3E*, *PIK3CB*, *TAB2*, *NFΚBIA*. 48 h after transfection of HEK 293 T cells with the indicated combinations of empty vectors, reporter gene constructs, empty expression plasmid pSG5 and miRNA-expression plasmids of miR-34a the cells were lysed and the luciferase activity was detected. The luciferase activity of the control vector experiments was set to 100%. The results represent the mean of four independent experiments carried out in duplicates. Three asterisks correspond to *p* < 0.001. Data are represented as mean ± SEM. **a** Results of dual luciferase assays with the *PLCG1*-3′UTR reporter plasmid (pMIR-RNL-TK-PLCG1-3′UTR). **b** Results of dual luciferase assays with the *CD3E*-3′UTR reporter plasmid (pMIR-RNL-TK-CD3E-3′UTR). **c** Results of dual luciferase assays with the *PIK3CB*-3′UTR reporter plasmid (pMIR-RNL-TK-PIK3CB-3′UTR). **d** Results of dual luciferase assays with the *TAB2*-3′UTR reporter plasmid (pMIR-RNL-TK-TAB2-3′UTR). **e** Results of dual luciferase assays with the *NFΚBIA*-3′UTR reporter plasmid (pMIR-RNL-TK-NFΚBIA-3′UTR)

To further explore the role of miR-34a in NF-κB signaling, we used miRWalk 2.0 to predict miR-34a target genes^20^. Thereby, we identified miR-34a binding sites in the 3’UTRs of 14 modulators of NF-κB including *CD3E*, *PLCG1*, *PIK3CB*, *MALT1*, *BCL10, TRAF2, TAB1, TAB2*, *TAK1*, *NFΚBIA, IKBKG*, *IKBKB*, *REL*, and *CREL*. The exact nucleotide positions of the predicted binding sites of miR-34a within the 3′UTRs of the five positive tested target genes is given in Fig. 1a–e. We amplified nucleotides 136–1200 of the 3′UTRs of *PLCG1*, nucleotides 1–573 of *CD3E*, nucleotides 1436–2137 of *PIK3CB*, nucleotides 102–1009 of *TAB2* and nucleotides 1–433 of *NFΚBIA*. The amplified sequences were cloned into the pMIR-RNL-TK reporter vector. HEK 293T cells were transfected with the miR-34a expression plasmid or the empty control vector and with reporter constructs harboring the predicted 3′UTRs or with empty reporter plasmids in the appropriate combinations (Fig. 2a–e).

The luciferase activity of the *PLCG1* reporter plasmid (pMIR-RNL-TK- PLCG1-3′UTR) was reduced to 70% (*p* < 0.001) as compared to pMIR-RNL-TK vector (Fig. 2a). The luciferase activity of the mutated *PLCG1* reporter plasmid was comparable to the activity of the empty pMIR-RNL-TK vector, verifying miR-34a binding to the predicted site. Likewise, the luciferase activities of the reporter plasmids for *CD3E*, *PIK3CB*, *TAB2*, and *NFΚBIA*, were each significantly reduced as compared to the pMIR-RNL-TK vector (Fig. 2b–e). In detail, the luciferase activity of *CD3E* reporter plasmid was reduced to 76%, the activity of *PIK3CB-* to 63%, the activity of *TAB2-* to 74%, and the activity of *NFΚBIA-*reporter vector to 53%. For each of the genes tested, the luciferase activity of the mutated reporter plasmid was comparable to the activity of the empty reporter vector and not significantly reduced.

For the remaining potential targets of miR-34a, we did not find a significant effect on the luciferase activity. In detail, we failed to provide evidence that the genes *BCL10*, *MALT1*, *TAK1*, *TAB1*, *TRAF2*, *IKBKG*, *IKBKB*, *REL*, and *CREL* are miR-34a targets (SFIG. 1A-J).

### Changes of endogenous expression of NFΚBIA as function of altered miR-34a levels

**Fig. 3:**
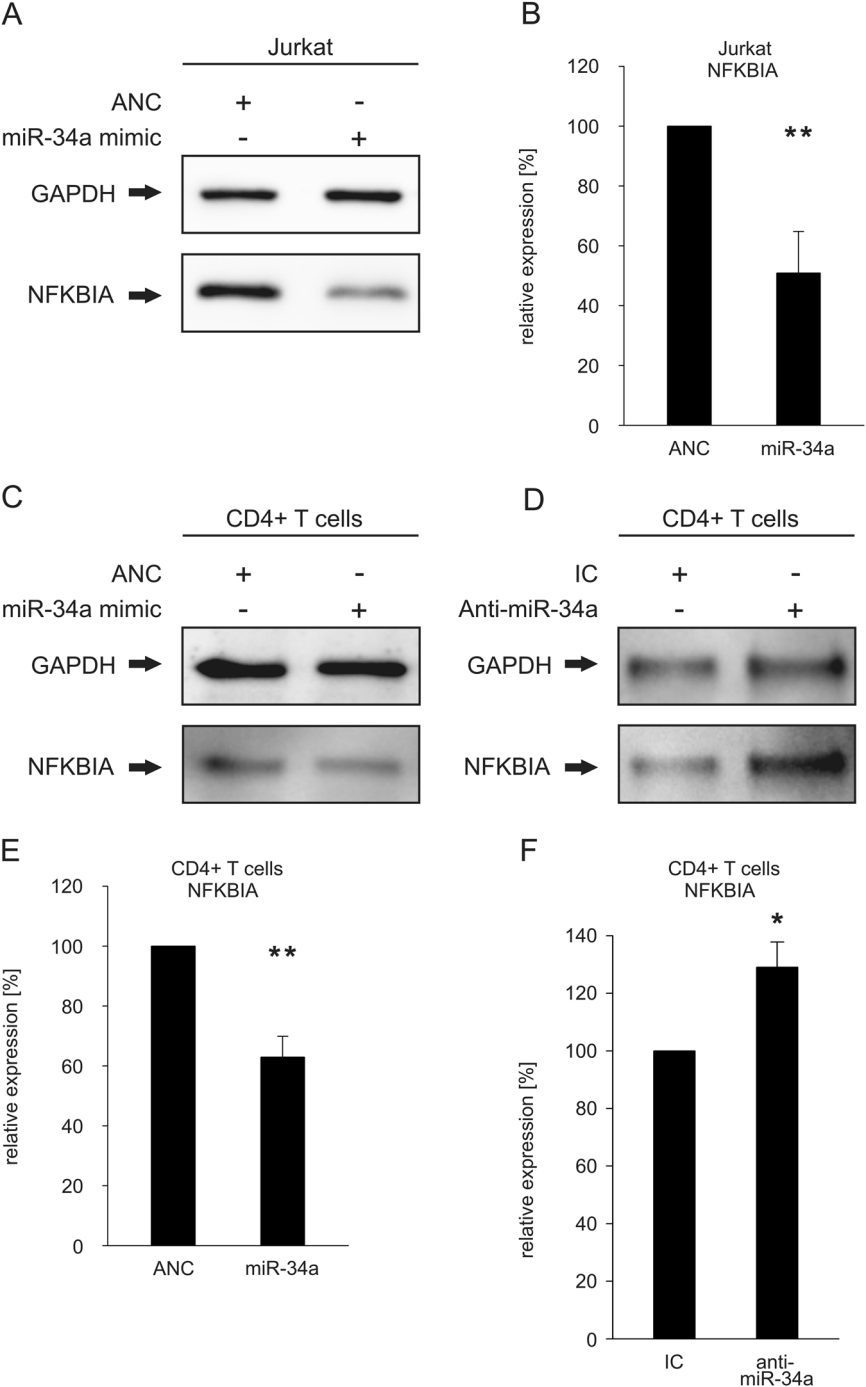
Regulation of the endogenous protein level of NFΚBIA by an altered miR-34a expression. **a** Western blot analysis of NFΚBIA in miR-34a-transfected Jurkat cells. Jurkat cells were transfected either with allstars negative control (ANC) or miR-34a-5p mimic. 48 h after transfection the endogenous protein level of NFΚBIA was analyzed by western blotting using specific antibodies against NFΚBIA. GAPDH served as loading control. **b** Quantification of NFΚBIA levels in miR-34a-transfected Jurkat cells. The expression of NFΚBIA in three independent western blot experiments was quantified by densitometry using Image Lab Software. The expression of NFΚBIA was normalized to the corresponding GAPDH signals of the respective samples. Two asterisks correspond to *p* < 0.01. **c**, **d** Analysis of the impact of altered miR-34a levels on the NFΚBIA protein level in CD4^+^ cells. CD4^+^ cells were transfected either with ANC or miR-34a-5p mimic (**c**) and with inhibitor control (IC) or anti-miR-34a-5p (**d**). 48 h after transfection the endogenous protein level of NFΚBIA was analyzed by western blotting using specific antibodies against NFΚBIA. GAPDH served as loading control. **e**, **f** Quantification of endogenous NFΚBIA levels in CD4^+^ T cells with altered miR-34a expression. The NFΚBIA protein expression in miR-34a transfected CD4^+^ T cells (**e**) and in anti-miR-34a transfected CD4^+^ T cells (**f**) was quantified by densitometry using Image Lab Software. Three independent Western blot experiments were quantified each. The expression of NFΚBIA was normalized to the corresponding GAPDH signals of the respective samples. One asterisks correspond to *p* < 0.05 and two asterisks correspond to *p* < 0.01. Data are represented as mean ± SD

**Fig. 4:**
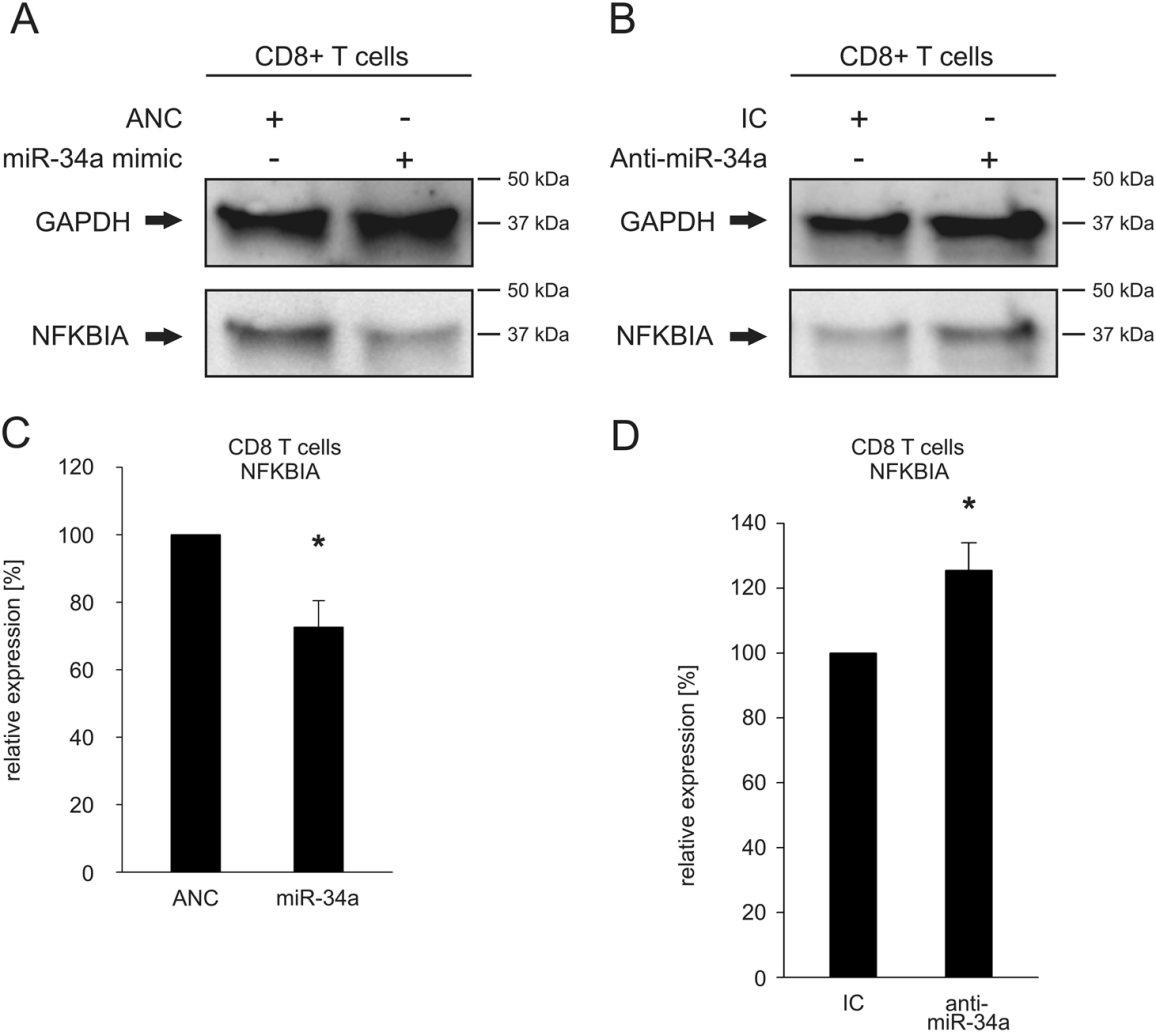
Regulation of the endogenous protein level of NFΚBIA by an altered miR-34a expression in CD8^+^ T cells. **a**, **b** Western blot analysis of the impact of altered miR-34a-levels on the endogenous NFΚBIA protein level in CD8^+^ cells. CD8^+^ T cells were transfected either with ANC or miR-34a-5p mimic (**a**) and with inhibitor control (IC) or anti-miR-34a-5p (**b**). 48 h after transfection, the endogenous protein level of NFΚBIA was analyzed by western blotting using specific antibodies against NFΚBIA. GAPDH served as loading control. **c**, **d** Quantification of endogenous NFΚBIA protein levels in CD8^+^ T cells with altered miR-34a expression. The NFΚBIA protein expression in miR-34a transfected CD8^+^ T cells (**c**) and in anti-miR-34a transfected CD4^+^ T cells (**d**) was quantified by densitometry using Image Lab Software. Three independent western blot experiments were quantified each. The expression of NFΚBIA was normalized to the corresponding GAPDH signals of the respective samples. One asterisks correspond to *p* < 0.05. Data are represented as mean ± SD

We next analyzed the effect of miR-34a on the endogenous NFKBIA protein. As readout system for the effects of miR-34a on the NF-κB pathway we choose NFΚBIA as the most downstream cytoplasmic member of the NF-κB pathway. We first transfected both Jurkat and primary CD4^+^ T cells with either “Allstars Negative Control (ANC)” as a non-targeting control or a miR-34a-5p mimic. The overexpression of miR-34a in the transfected CD4^+^ and CD8^+^ T cells of two different donors was confirmed by qRT-PCR as shown in supplementary figure 2 A-C. The hsa-miR-34a-5p mimic transfected CD4^+^ or CD8^+^ T cells showed elevated levels of hsa-miR-34a-5p in comparison to the controls (untreated cells (medium), mock transfected cells (HiPerFect) or with ANC transfected cells). Using a specific antibody against NFΚBIA, we analyzed the endogenous NFΚBIA levels by western blotting and detected reduced levels of NFΚBIA in both the miR-34a transfected Jurkat cells (Fig. 3a) and the transfected CD4^+^ T cells (Fig. 3c). A quantification of the NFΚBIA protein levels of three independent experiments showed that the mean NFΚBIA protein levels was decreased upon transfection of miR-34a to 50% (*p* < 0.01) in Jurkat cells (Fig. 3b) and to 69% (*p* < 0.01) in CD4^+^ cells (Fig. 3e). As a control experiment, we transfected CD4^+^ T cells with anti-miR-34a and found a significant increase of the NFΚBIA protein level to 129% (*p* < 0.05) providing further evidence for a functional relevance of miRNA-34a for the regulation of the NFΚBIA protein expression (Fig. 3f). Transfection of primary CD8^+^ T cells with a miR-34a-5p mimic likewise caused reduced levels of endogenous NFΚBIA (Fig. 4a). The mean NFΚBIA protein levels in CD8^+^ T cells were decreased to 72% (*p* < 0.05) (Fig. 4c). Transfection of CD8^+^ T cells with anti-miR-34a lead to an increase of 125% (*p* < 0.05) of the NFΚBIA protein level (Fig. 4b, d).

### Changes of the cell surface expression of TCRA and CD3E as function of altered miR-34a levels

**Fig. 5:**
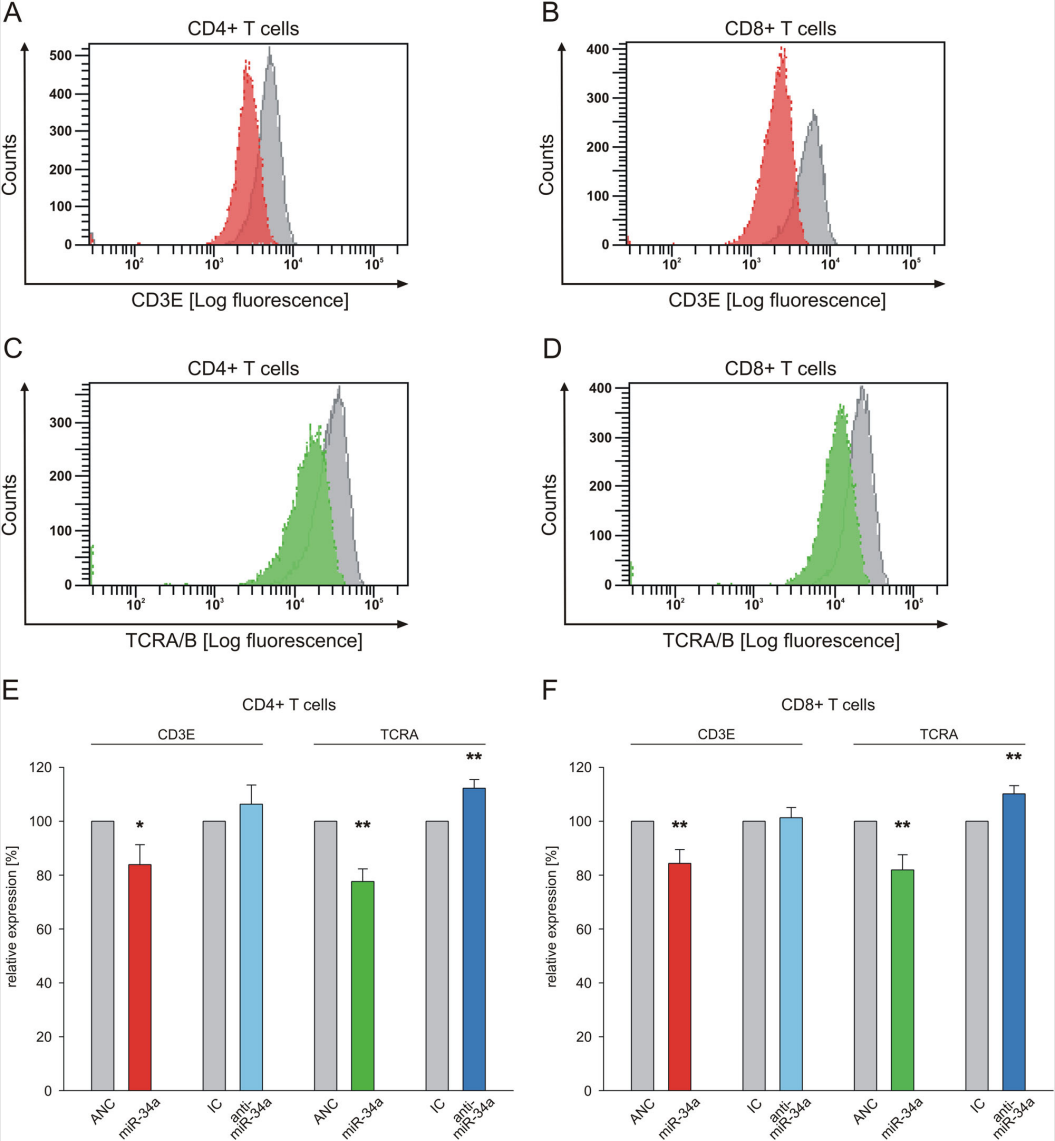
Changes of miR-34a expression in CD4^+^ and CD8^+^ T cells altered the cell surface expression of CD3E and TCRA. Primary CD4^+^ and CD8^+^ T cells were transfected either with nontargeting control (ANC allstars negative control) or miR-34a-5p mimic, respectively or with inhibitor control (IC) or anti-miR-34a-5p. **a**–**d** Mean fluorescence intensities of CD3E and TCRA expression, respectively from ANC-transfected (gray) or miR-34a-5p mimic-transfected (red and green, respectively) CD4^+^ or CD8^+^ T cells were analyzed. **e**, **f** FACS data of transfected primary CD4^+^ and CD8^+^ T cells were analyzed from three independent donor experiments performed in duplicates. One asterisk corresponds to *p* < 0.05 and two asterisks correspond to *p* < 0.01. Data are represented as mean ± SD

We next analyzed the effect of altered miR-34a levels in CD4^+^ and CD8^+^ T cells on the expression of CD3E and TCRA both of which map upstream of the NF-kB pathway using flow-cytometry (gating strategy is shown in SFIG. 3). Overexpression of miR-34a caused a reduction in the mean fluorescence intensities for CD3E and TCRA on CD4^+^ and CD8^+^ T cells in comparison to ANC-transfected cells. The respective changes are indicated in Fig. 5a–d in red for CD3E, green for TCRA and in gray for ANC-transfected cells. Quantification of three independent experiments from three different donors revealed a significant reduction in the cell surface expression of CD3E (84%; *p* < 0.05) and TCRA (78%; *p* < 0.01) on CD4^+^ T cells (Fig. 5e; CD3E: red, TCRA: green). Likewise, overexpression of miR-34a in CD8^+^ T cells led to a significant decrease of CD3E and TCRA cell surface levels to 84% (*p* < 0.01) and 81% (*p* < 0.01), respectively (Fig. 5f CD3E: red, TCRA: green). Inhibition of miR-34a in CD4^+^ T cells by transfection of anti-hsa-miR-34a-5p increased the cell surface level of CD3E to 107% (Fig. 5e; light blue) and of TCRA to 112% (*p* < 0.01) (Fig. 5e; dark blue). In CD8^+^ T cells inhibition of miR-34a increased the TCRA expression to 110% (*p* < 0.01) (Fig. 5f; dark blue) while the CD3E expression was not significantly affected in comparison to T cells transfected by inhibitor negative control (IC).

### Impact of miR-34a overexpression on cytotoxicity of CD8^+^ cells

**Fig. 6:**
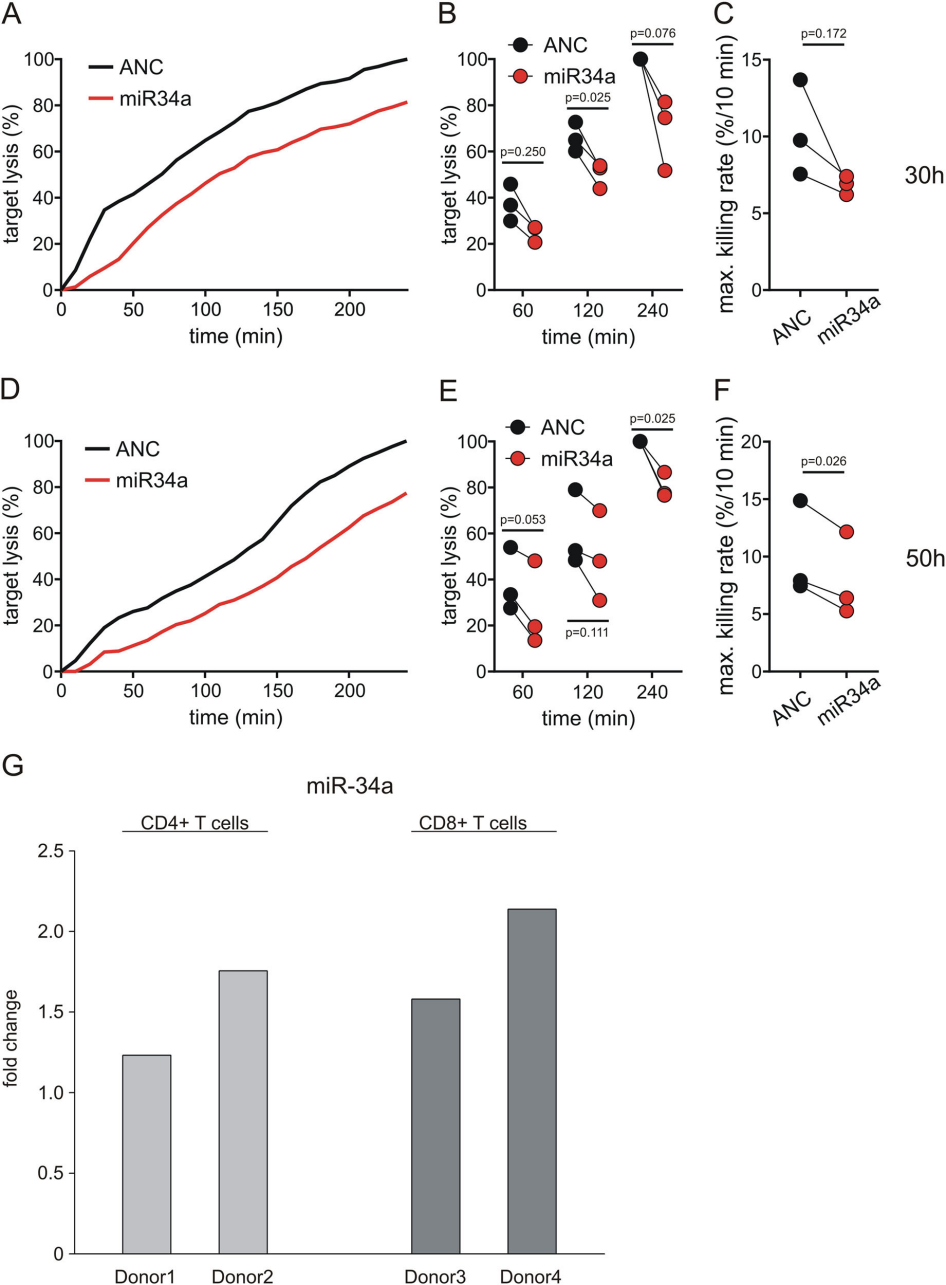
Killing efficiency in miR-34a-5p overexpressing MART1-specific CD8^+^ T cell clones. **a**, **d** Representative real-time killing assays in MART1-specific CD8^+^ T cell clones transfected with a miR-34a-5p mimic (red) or Allstars Negative Control (ANC) as control (black) using MART1 peptide-loaded T2 cells as target cells 30 h (**a**) and 50 h (**d**) after transfection. The effector cell to target cell ratio (E:T) was 2:1. **b**, **e** Target lysis at 60, 120, and 240 min analyzed in three independent experiments 30 h (**b**) and 50 h (**e**) after transfection (MART1-specific CD8^+^ T cell clones were independently expanded). **c**, **f** Quantification of maximal killing rates calculated from real-time killing kinetics (*n* = 3) 30 h (**c**) and 50 h (**f**) after transfection. qRT-PCR analysis of miRNA-34a expression in stimulated CD4^+^ and CD8^+^ T cells. **g** 7 h after activation of CD4^+^ and CD8^+^ cells from four different donors by CD2/CD3/CD28 beads the total RNA was isolated and miRNA-34a expression was analyzed by qRT-PCR using specific primers for miRNA-34a. The Fold change was calculated in reference to unstimulated medium controls

To analyze the impact of miR-34a on CD8^+^ T cell function, we investigated the effect of miR-34a overexpression on cytotoxicity in MART1-specific CD8^+^ T cell clones by a real-time killing assay^17^. CD8^+^ T cell clones were transfected with either ANC or a miR-34a-5p mimic and used as effector cells against MART1 peptide-loaded T2 target cells. Transfection efficiency and killing of miR-34a of transfected CD8^+^ T cell clones were measured 30 and 50 h after transfection by qRT-PCR and real-time killing assay, respectively. In this timeframe, transfection of miR-34a resulted in a 5–8-fold upregulation of miR-34a compared to ANC-transfected cells (SFig. 4). The upregulation of miR-34a induced a concomitant reduction in killing efficiency of transfected CD8^+^ T cell clones over the whole measurement periods (Fig. 6a, d). 30 h after transfection, the end point lysis (target cell lysis at timepoint 240 min of real-time killing assay) was reduced by at least 20% in miR-34a overexpressing CD8^+^ T cell clones (average end point lysis = 69.3%, normalized to ANC transfected CD8^+^ clones) (Fig. 6b). 50 h after transfection, the endpoint lysis of effector cells overexpressing miR-34a was reduced to 80.3% normalized to end point lysis of control cells (Fig. 6e). We further confirmed the impairment of cytotoxicity by quantifying the maximal killing rate (% target cell lysis/10 min). After 30 h, the maximal killing rate was reduced from 10.3%/10 min ±1.5 in ANC-transfected cells to 6.9%/10 min ± 0.3 in miR-34a transfected cells). After 50 h, the maximal killing rate was decreased from 10.1%/10 min ± 2.0 in ANC-transfected cells to 7.9%/10 min ± 1.7 in miR-34a-transfected cells.

### Rapid induction of miR-34a expression in TCR stimulated primary CD4^+^ and CD8^+^ T cells

To analyze the effect of NF-κB signaling on miR-34a expression, we stimulated CD4^+^ and CD8^+^ T cells, respectively, from four different donors with anti-CD2/CD3/CD28 coated beads. To confirm rapid T cell stimulation, p65 (RELA proto-oncogene, NF-kB subunit) and NFKBIA were analyzed. As expected, p65 expression was decreased in the cytoplasm and increased in the nucleus, and NFKBIA expression was decreased in the cytoplasm of CD4^+^ and CD8^+^ T cells (SFig. 5). After 7h of stimulation, we analyzed miR-34a expression by qRT-PCR. In stimulated CD4^+^ T cells the expression of miR-34a was increased by 1.2-fold for donor 1 and by 1.7-fold for donor 2. In stimulated CD8^+^ T cells the expression was increased by 1.6-fold in donor 3 and by 2.1-fold in donor 4 in reference to unstimulated controls (Fig. 6g).

## Discussion

Among the tested miRNA-34a target genes within the NF-κB signaling pathway in T cells, we identified TCRA, the alpha chain of the human TCR and CD3E, a subunit of the immunoreceptor-associated signal–transducing CD3 complex^21,22^, as direct targets of miRNA-34a (Table 1). The interaction of TCRA with a peptide-bound major histocompatibility complex molecule initiates the adaptive immune response^23^. As result of TCR ligand recognition conformational changes in the CD3E cytoplasmic tail are part of the earliest TCR signaling events upon antigen-binding to the TCR^24^. TCRA-deficient mice show an impaired Treg development and function^25^. CD3E deficiency caused by homozygous mutations in the CD3E gene is associated with the T^–^B^+^NK^+^ phenotype of severe combined immunodeficiency (SCID). In these patients no T cells are found in the peripheral blood indicating that the absence of CD3E completely inhibits human T cell differentiation^26^. Inhibition of CD3E affects the recruitment of the Src-family proteins tyrosine kinases, which phosphorylate tandem tyrosine residues within the immunoreceptor tyrosine-based activation motifs ITAMs^27^. Dually phosphorylated ITAMs lead to recruitment and activation of key downstream signaling molecules including the zeta chain of T-cell receptor-associated protein kinase 70 (ZAP70) in T cells^28^. The inhibition of CD3E, caused by miRNA-34a, may likewise impact the phosphorylation of ITAMS and the activation of ZAP70 that was also identified as direct target of miR-34a^29^. An increased expression of miR-34a is accompanied by reduced protein levels of ZAP70 resulting in less pronounced activation of downstream events^29^. One of the downstream targets of ZAP70 in the NF-κB signaling cascade is PRKCQ. In response to CD3/CD28 costimulation ZAP70 activates PRKCQ, which is required for NF-κB activation^30^. In our recent study, PRKCQ and other PKC-family members were identified as target genes of miR-34a^10^ suggesting that aberrant expression of miRNA-34a plays a role for T cells associated immunodeficiencies by interfering with the CD3E-ZAP70-PRKCQ axis.

**Table 1:** miRNA-34a target genes involved in TCR-NF-κB-signaling

Target gene	Aliases	Function	Mutation, gene deletion or silencing phenotype	Refs
*TCRA*	MD7, TCRD@, TRAC, TRA	Alpha chain of the T cell receptor	TCRA-/- deficient mice: defective thymic Treg selection	^25^
*CD3E*	IMD18, T3E, TCRE	Part of T cell receptor-CD3 complex: intracellular signal transduction	CD3E -/-: severe combined immunodeficiency with loss of T cells	^26^
*PLCG1*	NCKAP3, PLC-II, PLC1, PLC148, PLCgamma1	Catalyzes inositol 1,4,5-trisphosphate and diacylglycerol from phosphatidylinositol 4,5-bisphosphate.	activating mutations in adult T cell leukemia/lymphoma and angioimmunoblastic T cell lymphoma	^49, 32^
*PIK3CB*	P110BETA, PI3K, PI3KBETA, PIK3C1	Catalytic subunit of PIK3	PIK3CB -/- mice: early embryonic lethality	^33^
*TAB2*	CHTD2, MAP3K7IP2, TAB-2	IL-1-induced activation of NF-κB	TAB2 -/- mice: early embryonic lethality	^50, 35^
*NFKBIA*	IKBA, MAD-3, NFKBI	Inhibitor of NF-κB	NFKBIA -/- mice: severe hematological disorder and hypergranulopoiesis heterozygous mutation Ser 32: impaired NFKB translocation and T cell receptor mediated proliferation	^39, 40^

The miRNA-34a target PLCG1 generates diacylglycerol (DAG), which in turn phosphorylates PRKCQ leading to activation of NF-κB. In adult T cell leukemia (ATL) whole-exome sequencing identified 50 mutated genes including PLCG1 that was mutated in 36% of all investigated ATL cases^31^. Activating mutations of PLCG1 increasing the transcriptional activity of NF-κB via induction of MALT1 protease activity were also found in angioimmunoblastic T-cell lymphoma and other lymphomas derived from follicular T-helper cells^32^. The miRNA-34a target PIK3CB participates in TCR-mediated NF-κB signaling after binding of the costimulatory receptor CD28 by B7 ligands on antigen presenting cells (APCs)^13^. This molecular interaction activates PIK3 complex triggering phosphorylation of PRKCQ by pyruvate dehydrogenase kinase 1 leading to downstream activation of NF-κB^13^. Since PIK3CB knockout in mice is lethal at very early embryonic stages, T cells lacking PIK3CB are difficult to study^33^ (Table 1).

A further member of the TCR-NF-κB signaling and target of miRNA-34a is TAB2, which is ubiquitinated and degraded by RBCK1 (RANBP2-type and C3HC4-type zinc finger containing 1). By targeting TAB2 for degradation RBCK1 negatively regulates TAK1 (nuclear receptor subfamily 2 group C member 2) leading to NF-κB activation^34^. In mice the knockout of TAB2 has been linked to developmental defects and embryonic lethality^35^. Mutations in TAB2 are found in frontometaphyseal dysplasia causing increased TAK1 autophosphorylation and activation of NF-κB pathway^36^ (Table 1). The miR-34a target NFΚBIA inhibits translocation of NF-κB into the nucleus by masking the nuclear localization signal of NF-κB and retaining NF-κB as an inactive complex in the cytoplasm^37^. In response to immune or proinflammatory stimuli NFΚBIA is first phosphorylated and then ubiquitinated for degradation^38^. NFΚBIA−/− mice display a severe hematological disorder with an increase of granulocyte/erythroid/monocyte/macrophage colony-forming units (CFU-GEMM) and hypergranulopoiesis^39^. In humans, a heterozygous mutation of NFΚBIA at serine 32 of a patient with hyper immunoglobulin M-like immunodeficiency syndrome and ectodermal dysplasia was accompanied by an impairment of NF-κB translocation and of T cell receptor induced proliferation^40^ (Table 1). An inhibition of NFΚBIA translation by miR-34a combined with degradation of NFΚBIA by induction of NF-κB-signaling further enhances transcriptional activity of NF-κB.

Within the NF-κB signaling cascade phosphorylation is a crucial mechanism, which contributes to fast signal transduction thereby enabling T cells to an immediate response upon activation. Besides known proteins of the NF-κB signaling pathway like TCRA, CD3E, PLCG1, PIK3CB, TAB2, and NFΚBIA, miRNAs like miR-146, miR-155, and miR-34a emerge as a second layer of regulation of this pathway. In comparison to the protein phosphorylation, the slower kinetics of the miRNA-mediated regulation causes effects that likely are detectable only after several hours upon activation. Although binding of a miRNA to a mRNA target may result in an immediate decrease of the respective protein synthesis, a cellular effect is not to be expected as long as the total amount of protein as a function of the protein half live is not affected. In this respect we observed a reduction of cytoplasmic NFΚBIA and the subsequent increase of nuclear NF-κB four hours after T-cell activation.

Although the exact role of miR-34a in the NF-κB signaling pathway needs to be established, a possible scenario includes a positive feedback loop by which the amount of miR-34a is increased. An increased amount of miR-34a inhibits the translation of NFΚBIA, which in turn leads to an increase of nuclear NF-κB. This leads to a further activation of miR-34a transcription which is a known target of NF-κB^41^. This self-reinforcing process of miR-34a activation may also affect the other miR-34a targets within the NF-κB signaling pathway. The increasing amount of miR-34a progressively inhibits the targets TCRA, CD3E, PLCG1, PIK3CB, and TAB2, finally resulting in a “shutdown” of the NF-κB signaling process. In this scenario, the miRNA mediated regulation of the NF-κB pathway may be part of a mechanism that acts partly independent of canonical NF-κB signal transduction and contributes to a modulation of T cell activity. Parallel to initiating the canonical NF-κB signaling cascade, T cell activation triggers an increase of miRNA-34a expression resulting in a “temporary T cell inactivation” that may interrupt phases of T cell activity.

Increased understanding of the role of miRNA-34a in T cells depends on a better insight into the mechanisms of the cell killing including the processes terminating the interaction between T-cell and target cell and its kinetics/duration. Likewise, the role of miRNA-34a in naïve T cells needs to be clarified. The attenuated response of naïve T cells may be linked to altered NF-κB signaling, i.e., altered phosphorylation^42^, association with lipid rafts^43^, expression of signaling proteins^44–46^ and altered miRNA expression. Beside the role of miRNA-34a in these processes, miRNA-146 and miRNA-155 appear to play a pivotal role in regulation of T cell response during T cell activation^47,48^. Any final scenario describing the NF-κB regulation will have to acknowledge the role of miRNAs that are likely to be part of a still largely unknown layer of organization with a kinetic different from the canonical NF-κB signaling pathway.

Both by targeting multiple modulators of NF-κB signaling and by impairing CD8^+^ T cell-mediated cell killing, overexpression of miRNA-34a may play a central role in modulating T cell activation via a second layer of regulation with a kinetic different from the signal transduction by phosphorylation.

## Supplementary information

Supplementary Figures

Supplementary Table

Supplementary figure legends
