# Supplementary figures and images for: miR-34a: a new player in the regulation of T cell function by modulation of NF-κB signaling
Source: Cell Death Dis. 2019 Jan 18;10(2):46. doi: 10.1038/s41419-018-1295-1 (PMC6362007; doi:10.1038/s41419-018-1295-1)

SFig. 1

A

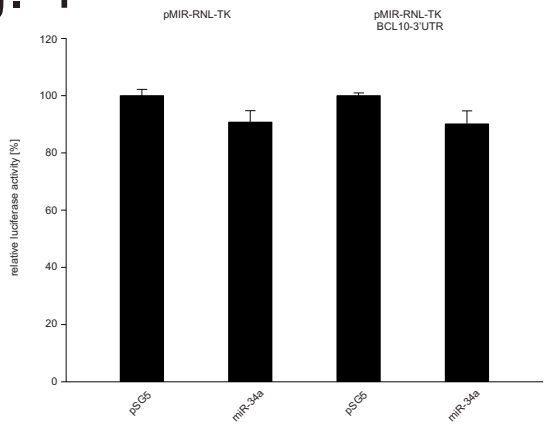

B

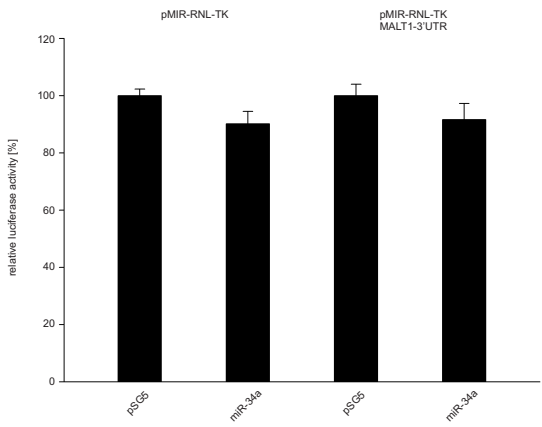

C

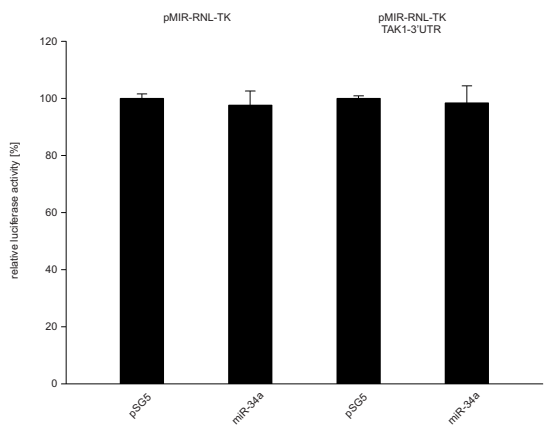

D

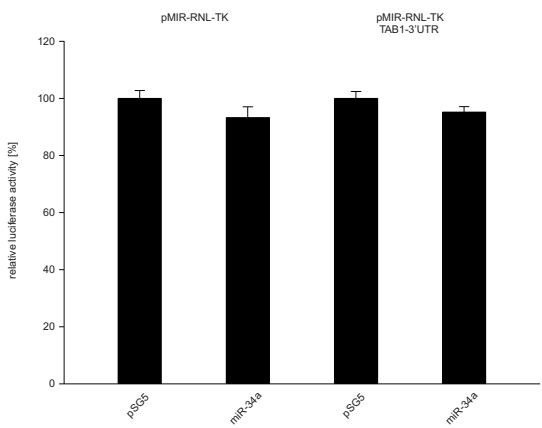

E

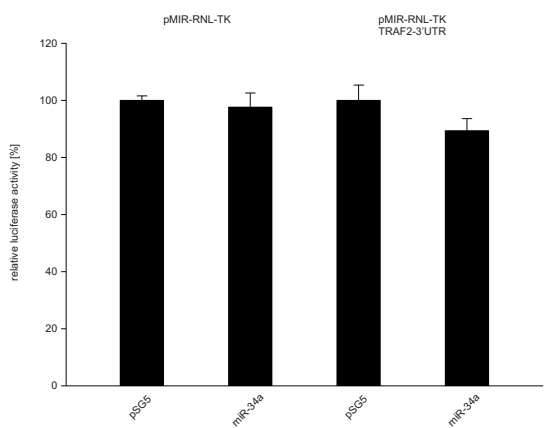

F

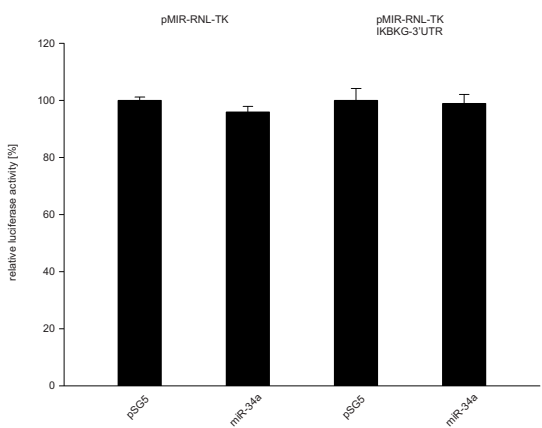

G

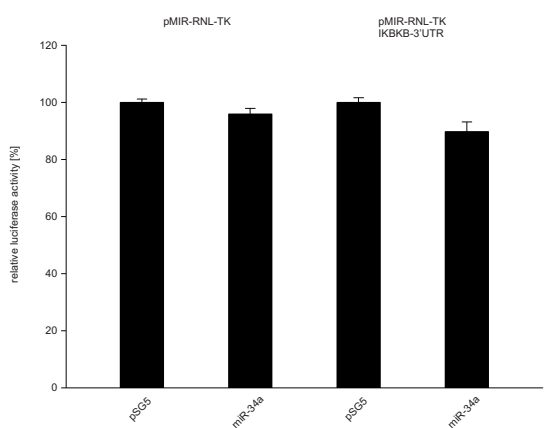

H

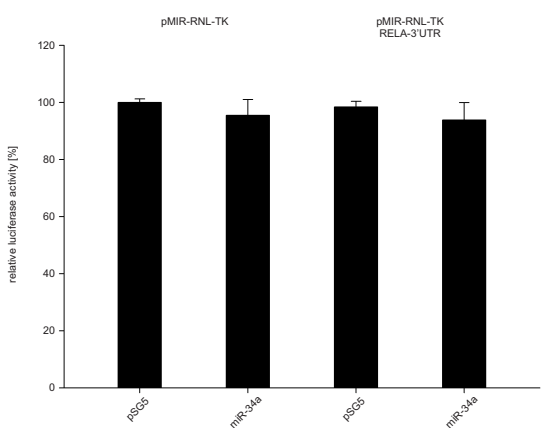

I

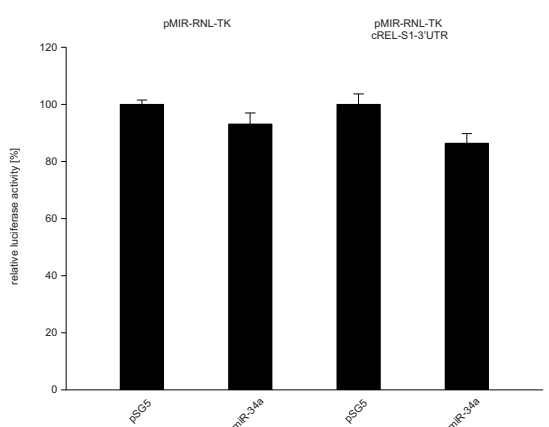

J

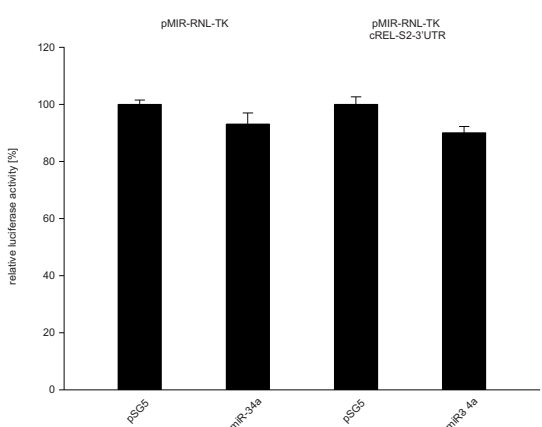

SFig. 2

A

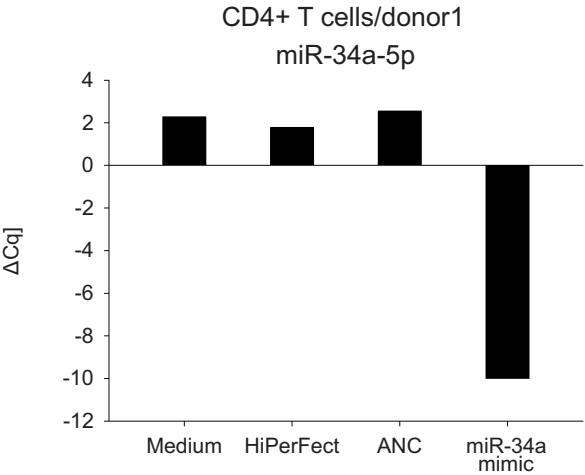

B

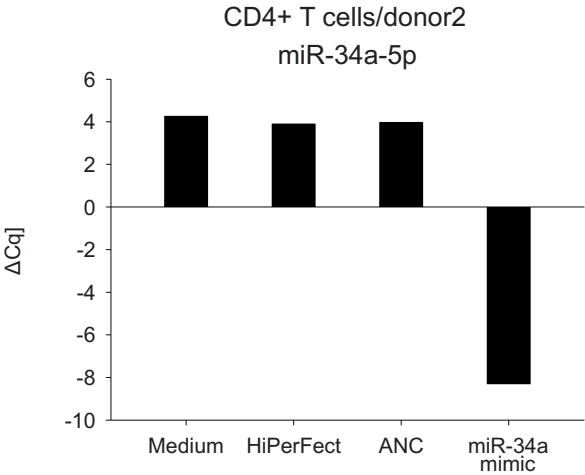

C

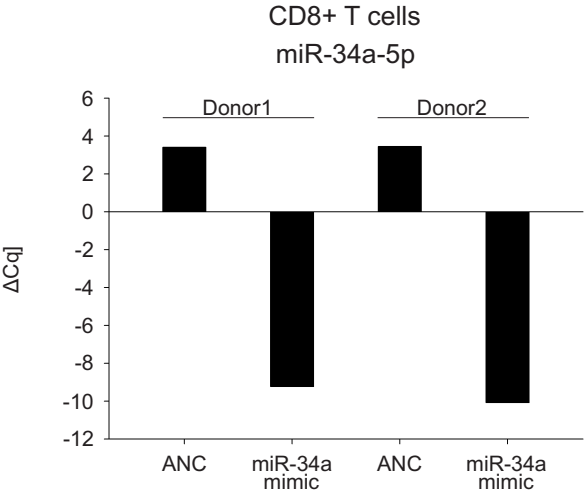

SFig. 3

A

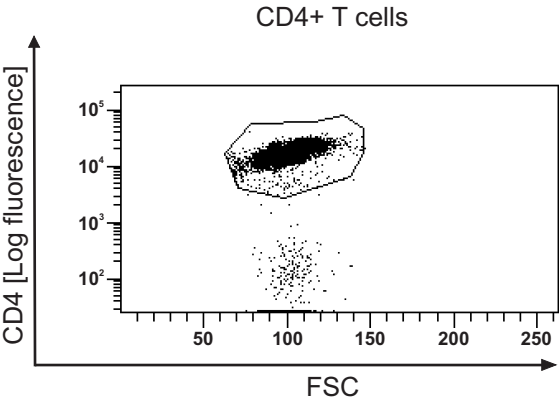

B

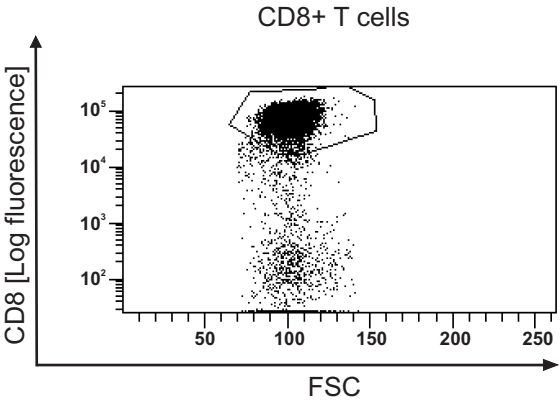

C

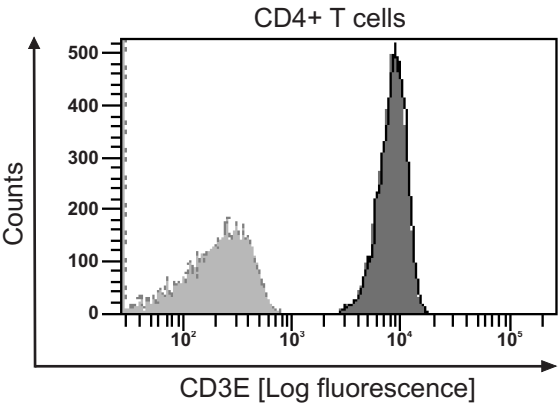

D

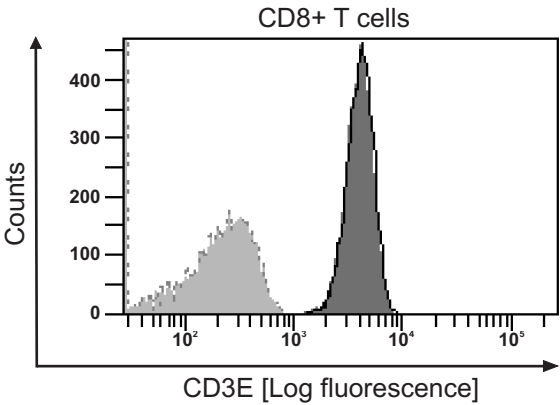

E

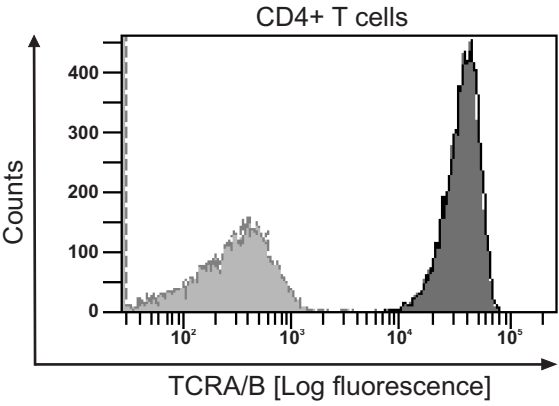

F

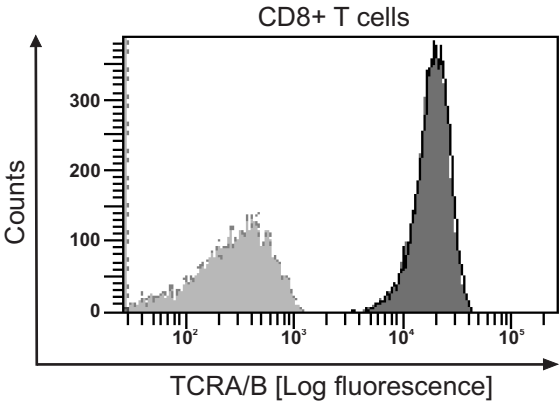

SFig. 4

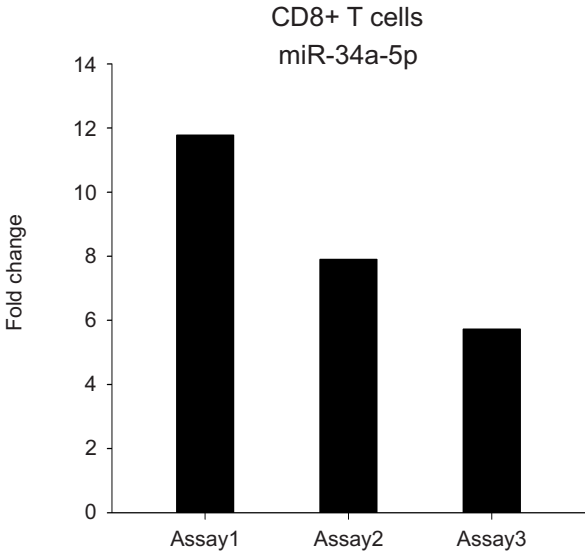

SFig. 5

A

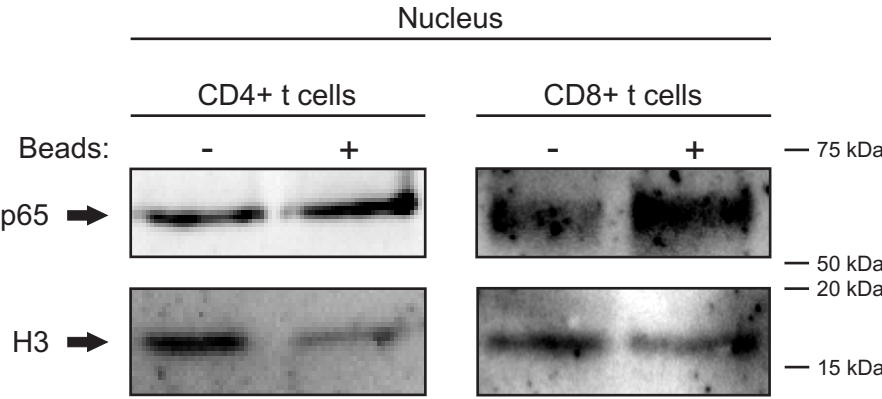

B

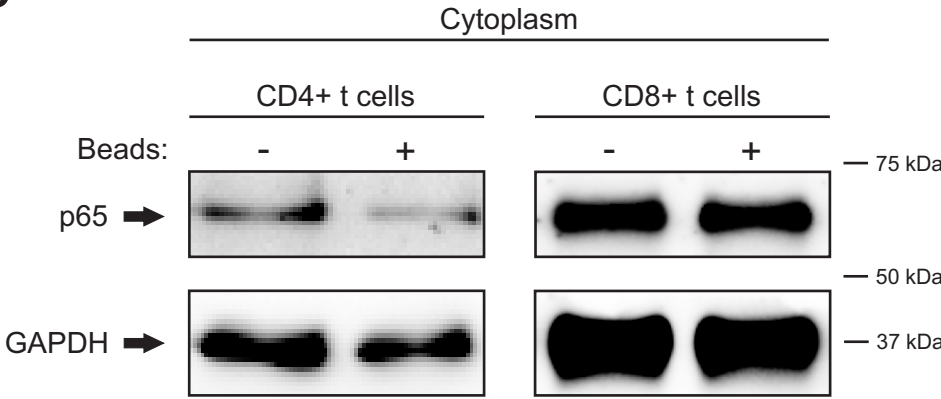

C

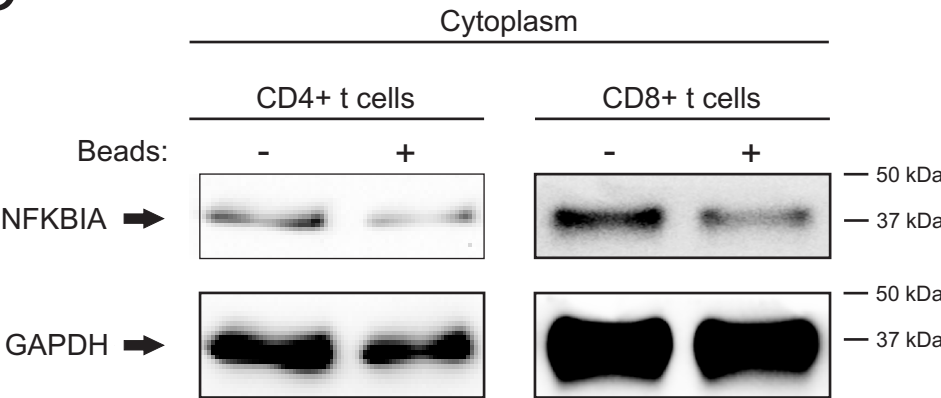

Supplement: Supplementary file 1 — Supplementary Figures [file 41419_2018_1295_MOESM1_ESM.pdf]
