# Supplementary material for: miR-34a: a new player in the regulation of T cell function by modulation of NF-κB signaling
Source: Cell Death Dis. 2019 Jan 18;10(2):46. doi: 10.1038/s41419-018-1295-1 (PMC6362007; doi:10.1038/s41419-018-1295-1)
Supplement: Supplementary file 2 — Supplementary Table [file 41419_2018_1295_MOESM2_ESM.pdf]

Supplementary Table 1

**cloning primers** **name**	*(restriction sites are underlined)* **sequence**
5'-cREL TS1-SpeI	ggactagtcagaaggcagttgaagtgag
3'-cRELTS1-SacI	cgagctcgaagagatgatggtggcttgg
5'-cREL TS2-SpeI	ggactagtgaactctacttgtgcactgg
3'-cREL TS2-SacI	cgagctccaggacattcaggtagcatg
5'-RELA-SpeI	ggactagtgaagccctccaaaagcacttac
3'-RELA-SacI	cgagctcgaaagagcaagagtccaagtg
5'-NFKBIA-SpeI	ggactagtgatgactgtgtgtttggagg
3'-NFKBIA-SacI	cgagctcgtacaccatttacaggaggg
5'-IKBKB-SpeI	ggactagtcgcttcctcagcagctgtgac
3'-IKBKB-SacI	cgagctcctagatgctgtgagagacag
5'-IKBKG-SpeI	ggactagtgcagatacatgtcatggagtg
3'-IKBKG-SacI	cgagctcgtccatcaatagtgactgg
5'-TAB1-SpeI	ggactagtcagtagagtgtgtgagtgcag
3'-TAB1-SacI	cgagctcggtactgtgtctcaaagaacc
5'-TAB2-SpeI	ggactagtgaaggtgtgacaagatggtg
3'-TAB2-SacI	cgagctccaaacactgtgttgtgtcacc
5'-TRAF2-SpeI	ggactagtcttcatcaaggccattgtgg
3'-TRAF2-SacI	cgagctccctaggaatgctcccttctctc
5'-BCL10-SpeI	ggactagtgacccttactaggaagaacg
3'-BCL10-SacI	cgagctcggaactccaaaatagcactcac
5'-PIK3CB-SpeI	ggactagtccaagtcagtagacaaaccaacc
3'-PIK3CB-NaeI	gccggcggcattgtgagtaaagagcacag
5'-MALT1-SpeI	ggactagtcacttcccaacttactctaggtc
3'-MALT1-SacI	cgagctccaactatgtatcaactgtgaatac
5'-PLCG1-SpeI	ggactagtgctagacagaaaccaagcc
3'-PLCG1-SacI	cgagctcaggatgatttatttggcagtcagatcttaagag<br>gg
5'-CD3E-SpeI	ggactagtcctgaatcagagacgcatc
3'-CD3E-SacI	cgagctcggagatgcaaatgaccatccag
**mutagenesis primers** **name**	*(mutated sites are shown in capital letters)* **sequence**
5'-CD3E-BS2mut	ggactagtcctgaatcagagacgcatctgaccctctggag<br>aaTCGCGAGtcccgctggcccagg
3'-CD3E-BS2mut	cctgggccagcgggaCTCGCGAttctccagagggtcagat<br>gcgtctctgattcaggactagtcc
5'-PIK3CB-mut	gtttggagaatggtgaaTCGCGAGaggaagaaatggatac
3'-PIK3CB-mut	gtatccatttcttcctCTCGCGAttcaccattctccaaac
5'-PLCG1-BS1mut	cagcacacacaaacctTCGCGAGcacatttgggctcag
3'-PLCG1-BS1mut	ctgagcccaaatgtgCTCGCGAaggtttgtgtgtgctg
5'-PLCG1-BS2mut	ccctcttaagatctgAGCGCTGaaataaatcatcctcgag<br>ctcg

3'-PLCG1-BS2mut	cgagctcgaggatgatttatttCAGCGCTcagatcttaag aggg
5'-TAB2-mut	gaaagacttggttgccTCGCGATtaactgtgtacagtg
3'-TAB2-mut	cactgtacacagttaaTCGCGAggcaaccaagtctttc
5'-NFKBIA-mut	cttatatccacactgcaTCGCGACtggcccaaaacgtc
3'-NFKBIA-mut	gacgttttgggccagTCGCGAtgcagtgtggatataag
