# Supplementary material for: miR-34a: a new player in the regulation of T cell function by modulation of NF-κB signaling
Source: Cell Death Dis. 2019 Jan 18;10(2):46. doi: 10.1038/s41419-018-1295-1 (PMC6362007; doi:10.1038/s41419-018-1295-1)
Supplement: Supplementary file 3 — Supplementary figure legends [file 41419_2018_1295_MOESM3_ESM.docx]

**SUPPLEMENTAL INFORMATION**

**Supplemental Figure Legends:**

**SFig. 1: Dual luciferase reporter assays of the negatively tested *BCL10*, *MALT1*, *TAK1, TAB1*, *TRAF2*, *IKBKG*, *IKBKB*, *RELA* and *cREL.*** 48h after transfection of HEK 293T cells with the indicated combinations of empty vectors, reporter gene constructs, empty expression plasmid pSG5 and miRNA-expression plasmids of miR-34a the cells were lysed and the luciferase activity was detected. The luciferase activity of the control vector experiments was set to 100%. The results represent the mean of four independent experiments carried out in duplicates. Data are represented as mean±SEM. **A:** Results of dual luciferase assays with the *BCL10-*3’UTR reporter plasmid, **B:** Results of dual luciferase assays with the *MALT1*-3’UTR reporter plasmid, **C:** Results of dual luciferase assays with the *TAK1*-3’UTR reporter plasmid, **D:** Results of dual luciferase assays with the *TAB1*-3’UTR reporter plasmid, **E:** Results of dual luciferase assays with the *TRAF2*-3’UTR reporter plasmid*,* **F:** Results of dual luciferase assays with the *IKBKG*-3’UTR reporter plasmid*,* **G:** Results of dual luciferase assays with the *IKBKB*-3’UTR reporter plasmid*,* **H:** Results of dual luciferase assays with the *RELA*-3’UTR reporter plasmid*,* **I:** Results of dual luciferase assays with the *cREL*-3’UTR segment1 reporter plasmid *,* **J:** Results of dual luciferase assays with the *cREL*-3’UTR segment2 reporter plasmid

**SFig. 2: Analysis of miR-34a-5p overexpression in CD4^+^ and CD8^+^ cells transfected with miR-34a-mimic using qRT-PCR.** CD4^+^ and CD8^+^ T cells were transfected either with “Allstars Negative Control (ANC)” or miR-34a-5p mimic. 48 h post transfection the total RNA was isolated and analyzed by qRT-PCR using a specific hsa-miR-34a-5p primer. The hsa-miR-34a-5p mimic transfected CD4^+^ or CD8^+^ T cells showed elevated levels of hsa-miR-34a-5p in comparison to the controls (untreated cells (medium), mock transfected cells (HiPerFect) or with ANC transfected cells).

**SFig. 3: FACS Controls**

CD4^+^ and CD8^+^ T cells were stained for CD4 or CD8, respectively and co-stained for CD3E, TCRA alpha/beta or the respective isotype controls for 30 min at 4°C. Cells were analyzed by flow cytometry. Gated CD4^+^ (A) or CD8^+^ (B) T cells were analyzed for CD3E (C,D dark grey) or TCRA alpha/beta (E, F, dark grey) expression in comparison to isotype controls (light grey).

**SFig. 4: qRT-PCR Killing Assays**

MART1-specific CD8^+^ T cell clones were transfected either with nontargeting control (allstars negative control=ANC) or miR-34a-5p mimic. 50 h post transfection the total RNA was isolated and analyzed by qRT-PCR using a specific hsa-miR-34a-5p primer.

**SFig.5: Western blot Analysis of activated CD4^+^ and CD8^+^ T cells.** 1x10^6^ CD4^+^ or CD8^+^ T cells were activated using CD28/CD2/CD3 beads. Four hours after activation the cells were lysed and the abundance of p65 and NFΚBIA in nucleus and cytoplasm was analyzed by Western blot using specific antibodies against p65 and NFΚBIA. H3 served as loading control in the nucleus and GAPDH in the cytoplasm protein extracts. **A:** Western blot analysis of p65 in the nucleus of CD4^+^ and CD8^+^ T cells. **B:** Western blot analysis of p65 in the cytoplasm of CD4^+^ and CD8^+^ T cells. **C:** Western blot analysis of NFΚBIA in the cytoplasm of CD4^+^ and CD8^+^ T cells.

**STab. 1: Sequences of cloning and mutagenesis primers**
